# Towards a precision approach to anesthetic/analgesic immunomodulation in cancer

**DOI:** 10.3389/fanes.2024.1464004

**Published:** 2024-12-05

**Authors:** Hersh V. Gupta, Kay S. Tan, Gregory W. Fischer, Joshua S. Mincer

**Affiliations:** 1 Department of Genetics, Albert Einstein College of Medicine, Bronx, NY, United States,; 2 Biostatistics Service, Department of Epidemiology and Biostatistics, Memorial Sloan Kettering Cancer Center, New York, NY, United States,; 3 Department of Anesthesiology and Critical Care Medicine, Memorial Sloan Kettering Cancer Center, New York, NY, United States,; 4 Department of Anesthesiology, Weill Cornell Medicine, New York, NY, United States

**Keywords:** anesthesia, analgesia, opioids, ketamine, ketorolac, immunomodulation, immunotherapy, precision medicine & genomics

## Abstract

**Background:**

Immunomodulation is widely invoked to explain possible effects of anesthetic/analgesic drugs on recurrence and survival in cancer patients. By analogy with immune checkpoint inhibitors, which enhance anti-tumor actions of immune cells in the tumor microenvironment (TME), we aim to develop a precision approach to immunomodulation by anesthetic/analgesic drugs. We explore biomarkers predictive of immunotherapy response [tumor mutational burden (TMB)] and resistance [fraction genome altered (FGA)] in relation to anesthetic/analgesic dose to survival response and the expression of drug target receptor genes.

**Methods:**

Two local clinical cohorts [lung adenocarcinoma (LUAD) and colon adenocarcinoma (COAD) patients] were analyzed retrospectively to yield statistical interactions between drugs, outcomes, and TMB/FGA (extending previously reported results). Bulk tumor gene expression data for solid tumors from 6,488 patients across 18 solid tumor types was obtained from The Cancer Genome Atlas (TCGA) and normalized by tumor type. TMB and FGA for each TCGA patient sample was extracted from cBioPortal. DeSeq was employed to quantify differential gene expression of target receptors of 79 common anesthetic/analgesic drugs for high/low TMB and FGA. Localization of these receptors to specific immune cells was estimated using CIBERSORT.

**Results:**

Increased TMB and FGA magnified opioid pro-tumor effects on overall survival in LUAD, while increased TMB reduced ketamine anti-tumor effects on recurrence and did not affect ketorolac anti-tumor effects on recurrence. In COAD, increased TMB (DNA mismatch repair deficiency) magnified opioid anti-tumor effects on recurrence. Drug target receptor gene expression (and immune cell-type specificity) correlated with both TMB and FGA as a function of cancer type.

**Conclusions:**

TMB and FGA may have utility as biomarkers predictive of individual cancer patient response to anesthetic/analgesic dose effects on survival due to immunomodulation. Correlation across cancer types of anesthetic/analgesic target receptor gene expression with TMB and FGA and with TME immune cell types suggests molecular/omics level targets for further mechanistic exploration. A precision oncoanalgesia approach in the cancer patient may ultimately be warranted to optimize oncological outcomes.

## Introduction

The question of whether anesthetic or analgesic drugs can have oncological effects remains very much incompletely answered (1) despite being raised over four decades ago (2). A recent line of inquiry maintains that the way to approach this question is from the vantage point of modern oncology (3, 4), to focus on the genomics (and transcriptomics, etc.) from which emerge the differences between cancer types generally and between individual patient tumors more specifically, and which are key determinants in the relationships between drug perturbations and oncological outcomes such as recurrence and survival (5).

Immunomodulation has long been invoked as the primary mechanism underlying anesthetic/analgesic drug effects on cancer (6). The specific formulation of this idea has historically been that of opioid-induced immunosuppression (7): opioids suppress immune function and enable tumor cells to escape immune-surveillance, leading to disease progression. The precision oncological approach has demonstrated the possibility that in fact opioids specifically may be either pro- or anti-tumor, depending on cancer type and variation in individual patient-specific tumor genomics, and furthermore that mechanisms underlying these effects may be immune-mediated as well as resulting from drug action on oncogenic pathways in tumor cells (8–13).

Ultimate validation of this precision approach is prediction of individual patient susceptibility to oncological effects of analgesic agents. Recent retrospective work integrating clinicopathologic and omics data has generated hypotheses for specific tumor genomic mutations that may be able to predict opioid, ketamine, and ketorolac effects on oncological outcomes for specific patients, pending further prospective validation, through direct on-tumor effects (11–13). The aim of the present work is to identify factors that may do the same based on immune-mediated effects of analgesic agents, i.e., to articulate a precision approach to analgesic-induced immunomodulation.

From the oncological viewpoint, an analogy can be made between anesthetic/analgesic drugs and immunotherapeutic agents, in particular immune checkpoint inhibitors (ICI), which enhance anti-tumor actions of immune cells in the tumor microenvironment (TME) (14). Recognizing that a particular anesthetic/analgesic drug may be pro- or anti-tumor depending on the context (cancer type and individual patient-specific tumor omics), the common factor here is immunomodulation at the cellular level, i.e., action on immune cells in the TME. As with ICI, characteristics of the TME may render a specific patient’s tumor more susceptible to drug effects on TME immune cells. Practically, specific biomarkers may reflect these characteristics and enable prediction of drug effects.

The fundamental characteristic determining ICI efficacy is tumor immunogenicity, which can be defined as a tumor’s ability to induce anti-tumor immune response (15), reflected by high levels of tumor-infiltrating lymphocytes (TILs). A major contributor to immunogenicity is tumor mutational burden (TMB), defined as the number of somatic mutations per megabase of genomic sequence (16). TMB captures the number of non-silent mutated single base pairs in tumor DNA, meaning that these mutations result in tumor cells producing neoantigens, i.e., new molecules translated from the mutated DNA that bind to T-cell receptors, are recognized as “non-self”, and therefore stimulate anti-tumor immune activity (17). TMB has been extensively studied as a predictive biomarker for response to ICI (18, 19) and is used clinically to guide ICI treatment (20), though challenges remain in terms of its general implementation across cancer types and individuals (21).

We hypothesize that immunomodulation by anesthetic/analgesic drugs should similarly depend on tumor immunogenicity since these drugs need to have a substrate (i.e., TILs) to modulate to exert on-tumor effects (either pro- or anti-tumor). We therefore hypothesize that TMB may serve as a predictive biomarker for individual patient susceptibility to immunomodulation by these drugs, manifesting as a change in the dose-response curves for associations between drug dose and oncological outcome. We further explore anesthetic/analgesic target receptor gene expression in relation to individual patient immunogenicity, as represented by TMB. In addition to TMB, we explore dose-response and receptor gene expression in the context of another ICI biomarker, fraction genome altered (FGA), which measures copy number variation (deletion or new copies of entire genome segments). FGA reflects duplication or deletion of DNA in lengths from single genes to entire chromosomes, in contrast to the single base pair changes captured by TMB, and in contrast to TMB appears to predict resistance to ICI (22–24).

## Methods

### Patient cohorts: MSK

We previously retrospectively analyzed two large prospectively collected datasets for early-stage patients presenting for primary tumor resection at Memorial Sloan Kettering Cancer Center: 740 patients with lung adenocarcinoma (MSK-LUAD) (11) and 1,157 patients with colon adenocarcinoma (MSK-COAD) (12). In the present work we revisited these datasets to further probe relationships between TMB, FGA, drugs, and outcomes.

### Patient cohorts: The Cancer Genome Atlas (TCGA)

The Cancer Genome Atlas (TCGA) (16) contains clinicopathologic and bulk RNA sequencing gene expression data for over 20,000 primary cancer samples from 33 cancer types. We focused on 18 TCGA solid tumor cohorts, corresponding to 6,488 patients (see Supplementary Table S1 for a breakdown of total samples by cancer type).

### Categorization of TMB and FGA into clinically significant groupings

The MSK-LUAD cohort featured next-generation sequencing (MSK-IMPACT) (25) of tumor samples removed during surgery, which enabled calculation of TMB and FGA (among other genomic factors). The MSK-COAD cohort featured immunohistochemical determination of DNA mismatch repair (MMR) status [either deficient (dMMR) or proficient (pMMR)] (26). MMR status correlates with TMB, where dMMR patients have high TMB and pMMR patients have low TMB (27, 28). TCGA cohorts featured TMB and FGA values for patient tumor samples, which were obtained through cBioPortal (29–31). TMB in TCGA was determined by the total number of nonsynonymous coding mutations.

Distributions for TMB and FGA were calculated separately for the MSK and TCGA cohorts by plotting log TMB or unnormalized FGA vs. TMB/FGA rank. Since TMB distributions are known to differ by cancer type (32), we calculated separate distributions for the different TCGA cohorts. Consistent with existing literature and clinical algorithms for immunotherapy response prediction (33, 34), these distributions were not linear but rather featured breakpoints dividing the distributions into high (hypermutators) (35) and low TMB and high, mid, and low FGA. Three breakpoints were computed for TMB, while two were computed from FGA. Because TMB was binarized, the choice between the 2nd and 3rd breakpoints was manual; the 1st breakpoint was discarded as it represented samples with virtually no mutations and so was too low to be the hypermutation breakpoint. Breakpoints were calculated using the R package Segmented (36) with the mixed-linear segmented models.

### Calculation of predicted survival estimates in the MSK cohorts

The multivariable regression models developed previously (11–13) were employed to calculate predicted 5-year survival estimates for model patients for specific TMB and FGA values.

### Bulk RNA sequencing gene expression data and analysis

Bulk RNA sequencing data was analyzed for the same TCGA patients described above. HTSeq files containing raw RNA counts were downloaded from the National Institutes of Health/National Cancer Institute Genomic Data Commons public database (37). Standard DESeq2 workflow (38) was employed to normalize the data and calculate differential gene expression for the TMB and FGA categories described above (i.e., high vs. low TMB and high vs. low FGA). In brief, genes were prefiltered by cancer to only include genes where at least 50% of the samples had non-zero count data. Standard differential expression using DESeq2 was carried out and log_2_ fold-change estimate shrinkage was carried out using the APEGLM method (39). *P*-values were automatically adjusted in DESeq2 for multiple testing correction. Count data was normalized using the variance-stabilized transform algorithm, which decreases the variance of counts as compared to the mean of counts for a given gene, along with outputting expression in log scale.

### Anesthetic/analgesic target receptor genes

A list of 79 genes corresponding to target receptors (or subunits where applicable) for common anesthetic and analgesic drugs was compiled, listed in Supplementary Table S2.

### Computational estimation of TILs

Computational estimates for immune cell-type proportions for each patient sample in the TCGA cohorts were previously computed by Wang and colleagues (40) using the default LM22 immune composition expression matrices (41) in CIBERSORT (42).

## Results

### TMB and FGA modify anesthetic/analgesic dose to survival response curves

Increased intraoperative opioid dose was previously found to be associated with worse overall survival (OS) in the MSK-LUAD cohort (11), while intraoperative ketamine (vs. either dexmedetomidine or no adjunct) (11) and intraoperative ketorolac were associated with higher recurrence-specific survival (RSS) (13). TMB was previously found to interact with opioid dose to modify the opioid-OS association (11), though not with ketorolac (13). Further analysis of this dataset in this work demonstrated that TMB interacted with ketamine to modify the ketamine-RSS association (see below). FGA was previously found to modify the opioid-OS association (11).

**FIGURE 1:**
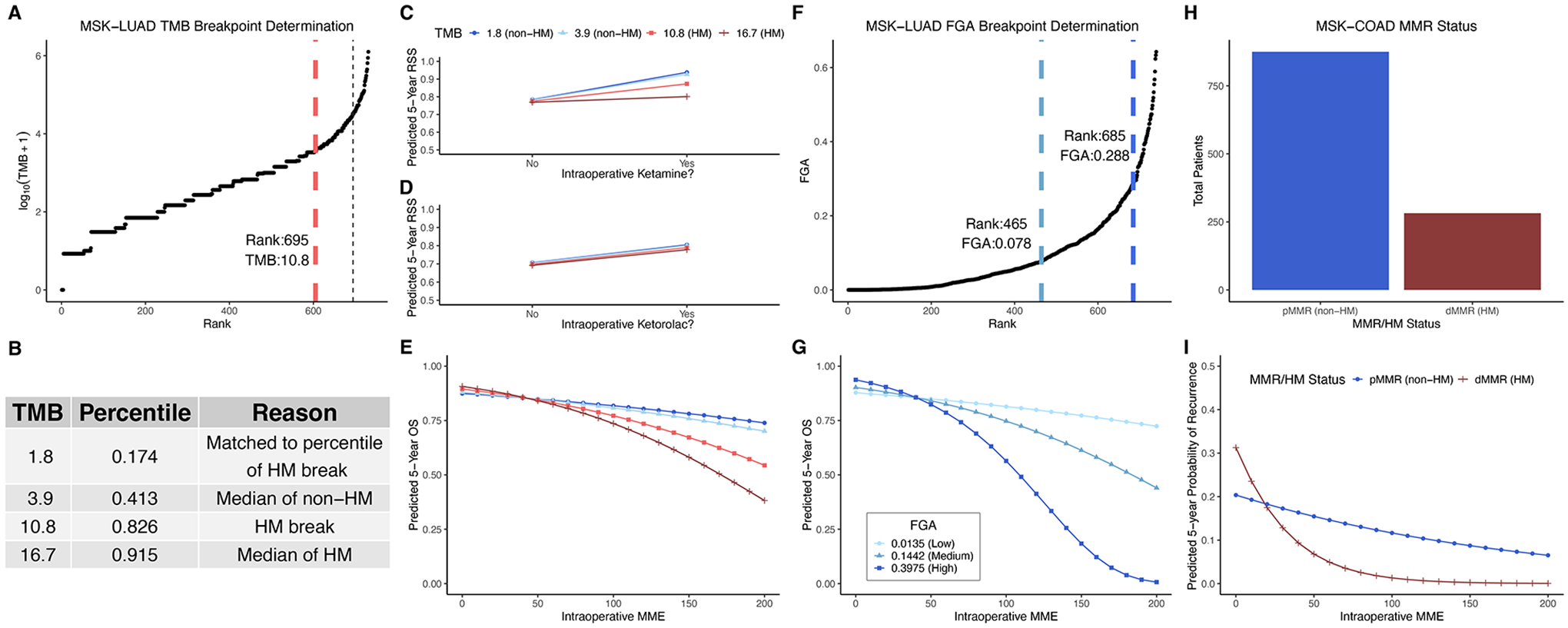
TMB and FGA modify anesthetic/analgesic dose to survival response curves in two cancer types: TMB distribution for the MSK-LUAD cohort **(A)**; predicted 5-year survival estimates for model patients with derived TMB values **(B)** plotted for RSS for ketamine vs. no adjunct **(C)**, RSS for ketorolac vs. no ketorolac **(D)**, and OS vs. opioid dose **(E)**; FGA distribution for the MSK-LUAD cohort **(F)**; predicted 5-year OS estimates across a range of opioid doses for model patients with derived FGA values **(G)**; MMR status distribution for the MSK-COAD cohort **(H)** and predicted 5-year OS estimates across a range of opioid doses for model patients based on MMR status **(I)**. FGA, fraction genome altered; HM, hypermutator (high TMB); MME, oral morphine milligram equivalents; MMR, DNA mismatch repair (pMMR, MMR-proficient; dMMR, MMR-deficient), MSK-COAD, Memorial Sloan Kettering colon adenocarcinoma cohort; MSK-LUAD, Memorial Sloan Kettering lung adenocarcinoma cohort; OS, overall survival; RSS, recurrence-specific survival; TMB, tumor mutational burden.

To probe these interactions further, we calculated predicted 5-year survival estimates for model patients with different TMB values, derived from the TMB distribution for MSK-LUAD cohort (Figure 1A) as explained in Figure 1B (TMB values: 1.8 (17th percentile), 3.9 (41st percentile), 10.8 (83rd percentile), and 16.7 (92nd percentile)). The 10.8 muts/mb cutoff separating hypermutators from non-hypermutators is consistent with the FDA-defined cutoff (10 muts/mb) above which patients are considered hypermutators and approved to receive pembrolizumab (20). TMB interacted with ketamine such that increased TMB [hypermutation (HM)] opposed ketamine’s effect to reduce recurrence (Figure 1C, TMB = 1.8: predicted 5-year RSS = 0.94 with ketamine vs. 0.78 for no adjunct; 3.9: 0.93 vs. 0.78; 10.8: 0.87 vs. 0.78; 16.7: 0.80 vs. 0.77). TMB did not significantly interact with ketorolac, illustrated by the overlapping curves for TMB values (Figure 1D, TMB = 1.8: predicted 5-year RSS 0.81 with ketorolac vs. 0.71 without; 3.9: 0.80 vs. 0.71; 10.8: 0.79 vs. 0.70; 16.7: 0.78 vs. 0.69). TMB increase magnified the opioid-OS association such that OS further worsened at higher opioid dose (Figure 1E).

For FGA, the median of each group (high, mid, and low) was selected from the distribution (Figure 1F) for further analysis; these were 0.0135 (31st percentile), 0.1442 (78th percentile) and 0.3975 (96th percentile). Like TMB, FGA increase magnified the opioid-OS association such that OS further worsened at higher opioid dose (Figure 1G). Note that the error on these predicted values increases for higher opioid dose and FGA (Supplementary Figure S1) owing to the decreasing number of patients at both higher MME and high FGA, and so the observed near 100 percent mortality at high FGA and high opioid dose should be interpreted in that context, with a focus on the trend of increasing mortality for a given opioid dose as FGA increases.

In the MSK-COAD cohort, increased intraoperative opioid dose was associated with lower risk of recurrence (12) (highlighting that oncological effects of opioids may depend on cancer type/subtype). For this analysis, a correlate of TMB, MMR status, was used to determine hypermutation high-TMB status (Figure 1H). Hypermutation (dMMR) further decreased recurrence at higher opioid dose. (Figure 1I, modified from the original figure).

### Anesthetic/analgesic target receptor gene expression is correlated with TMB and FGA

**FIGURE 2:**
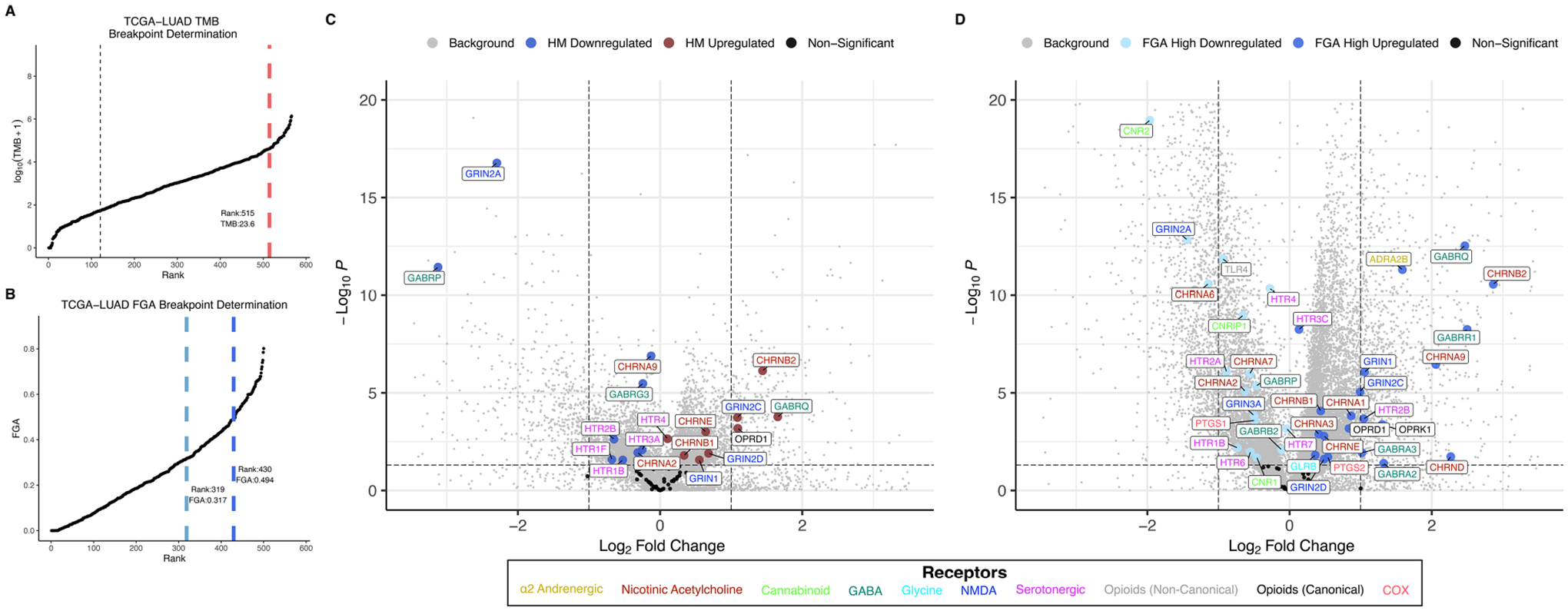
TMB and FGA modify expression of anesthetic/analgesic target receptor genes in lung adenocarcinoma: Distributions for TMB **(A)** and FGA **(B)** and differential gene expression for high vs. low TMB **(C)** and FGA **(D)** plotted for the TCGA-LUAD cohort (horizontal line denotes *p* = 0.05, vertical lines denote fold change = ±1 left and right, respectively). FGA, fraction genome altered; HM, hypermutator (high TMB); TCGA-LUAD, The Cancer Genome Atlas lung adenocarcinoma cohort; TMB, tumor mutational burden.

**FIGURE 3:**
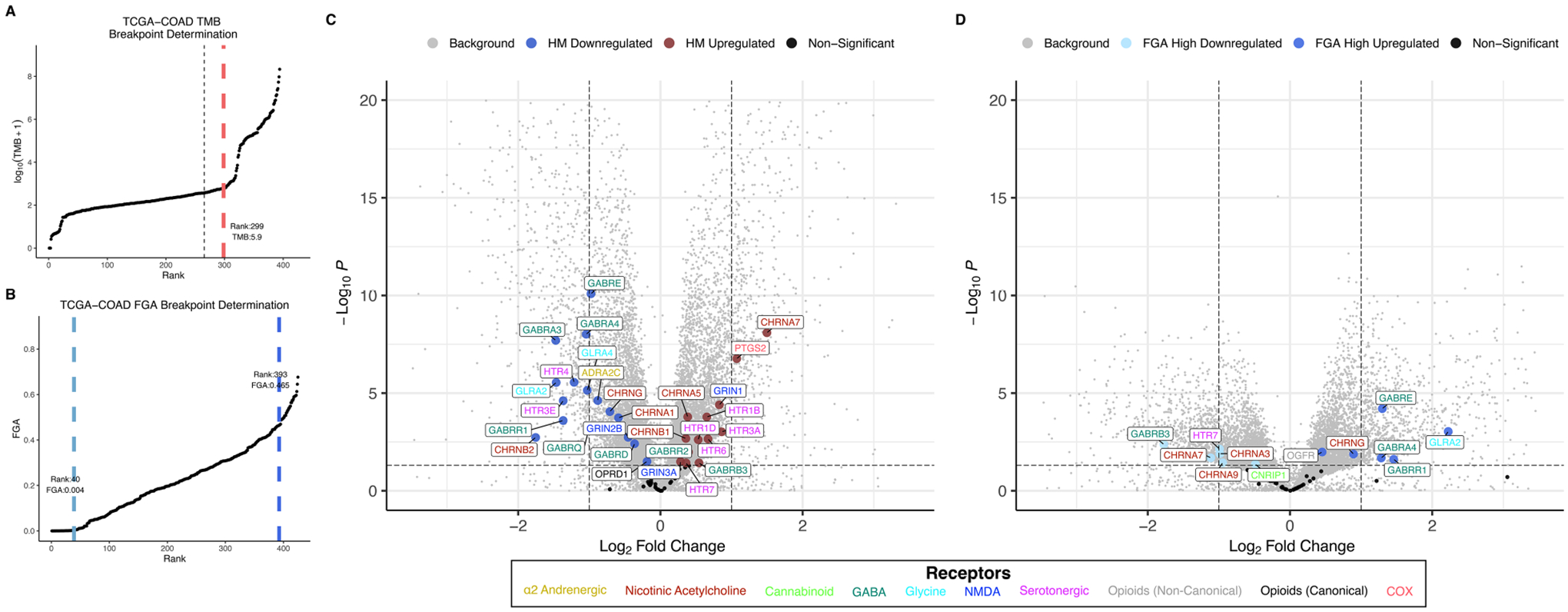
TMB and FGA modify expression of anesthetic/analgesic target receptor genes in colon adenocarcinoma: Distributions for TMB **(A)** and FGA **(B)** and differential gene expression for high vs. low TMB **(C)** and FGA **(D)** plotted for the TCGA-COAD cohort (horizontal line denotes *p* = 0.05, vertical lines denote fold change = ±1 left and right, respectively). FGA, fraction genome altered; HM, hypermutator (high TMB); TCGA-COAD, The Cancer Genome Atlas colon adenocarcinoma cohort; TMB, tumor mutational burden.

Distributions for TMB and FGA by cancer type are shown for the TCGA lung adenocarcinoma cohort (TCGA-LUAD) in Figures 2A,B and for the TCGA colon adenocarcinoma cohort (TCGA-COAD) in Figures 3A,B (and for all 18 TCGA cancer types in Supplementary Figure S2, S3 for TMB and FGA respectively with breakpoints provided in Supplementary Table S3, S4 for TMB and FGA respectively). For TCGA-LUAD, the breakpoint separating high and low TMB was 23.57 muts/mb. The breakpoints separating low to mid FGA and mid to high FGA were 0.318 and 0.494, respectively. For TCGA-COAD, the TMB breakpoint was 5.9 muts/mb, while the breakpoints separating low to mid FGA and mid to high FGA were 0.004 and 0.465, respectively. Note that these values are determined from whole-exome sequencing and do not correspond exactly to those calculated from a targeted oncology panel like MSK-IMPACT [the harmonization of different panel assays and whole exome sequencing is the subject of ongoing research (43, 44)].

Differential gene expression for TCGA-LUAD is illustrated in the volcano plot Figure 2C for high vs. low TMB and in Figure 2D for high vs. low FGA. For the TMB differential expression, 6,469 of a total 31,521 genes (20.5%) were significantly differentially expressed, with 18 of the 59 expressed anesthetic receptor genes (30.5%) being differentially expressed. For the FGA differential expression, 16,082 total genes were differentially expressed (51.0%) with 38 of anesthetic target receptors (64.4%) being differentially expressed. Differential gene expression for TCGA-COAD is shown similarly in Figures 3C,D. For TCGA-LUAD, 12,492 of 28,711 (43.5%) total genes were found to be differentially expressed for TMB with 32 of the 58 (55.2%) expressed anesthetic receptors being differentially expressed. In the FGA comparison, 7,479 (26.0%) of all genes were differentially expressed, and 12 of the 58 (20.7%) of the anesthetic genes had differential expression. Note that while this demonstrates overrepresentation of the receptor genes, more relevant to the current analysis is that there is differential expression of these genes (and the specific genes that are differentially expressed).

Anesthetic/analgesic target receptor genes are highlighted throughout. In TCGA-LUAD, an increase in TMB or FGA generally causes a subset of same genes to be upregulated (e.g., *OPRD1, GRIN2D, GRIN1, GRIN2C*) and downregulated (e.g., *GRIN2A, GABRP, CHRNA2)*, though some may switch, such as *CHRNA9*. However, in TCGA-COAD, most of the genes switch up- or downregulation when comparing an increase in TMB vs. FGA (e.g., *GABRB3, HTR7, CHRNA7, GABRE, CHRNG, GLRA2*). It should also be noted that FGA for TCGA-LUAD and TMB for TCGA-COAD showed an increase in differential expression in both the background genes, as well as the target receptor genes, as compared to TMB and FGA, respectively. As a check, differential expression between FGA medium and high was carried out for TCGA-COAD and TCGA-LUAD (Supplementary Figure S4). This showed in general less differential expression than the FGA high vs. low comparisons, and no additional genes were found to be enriched.

**FIGURE 4:**
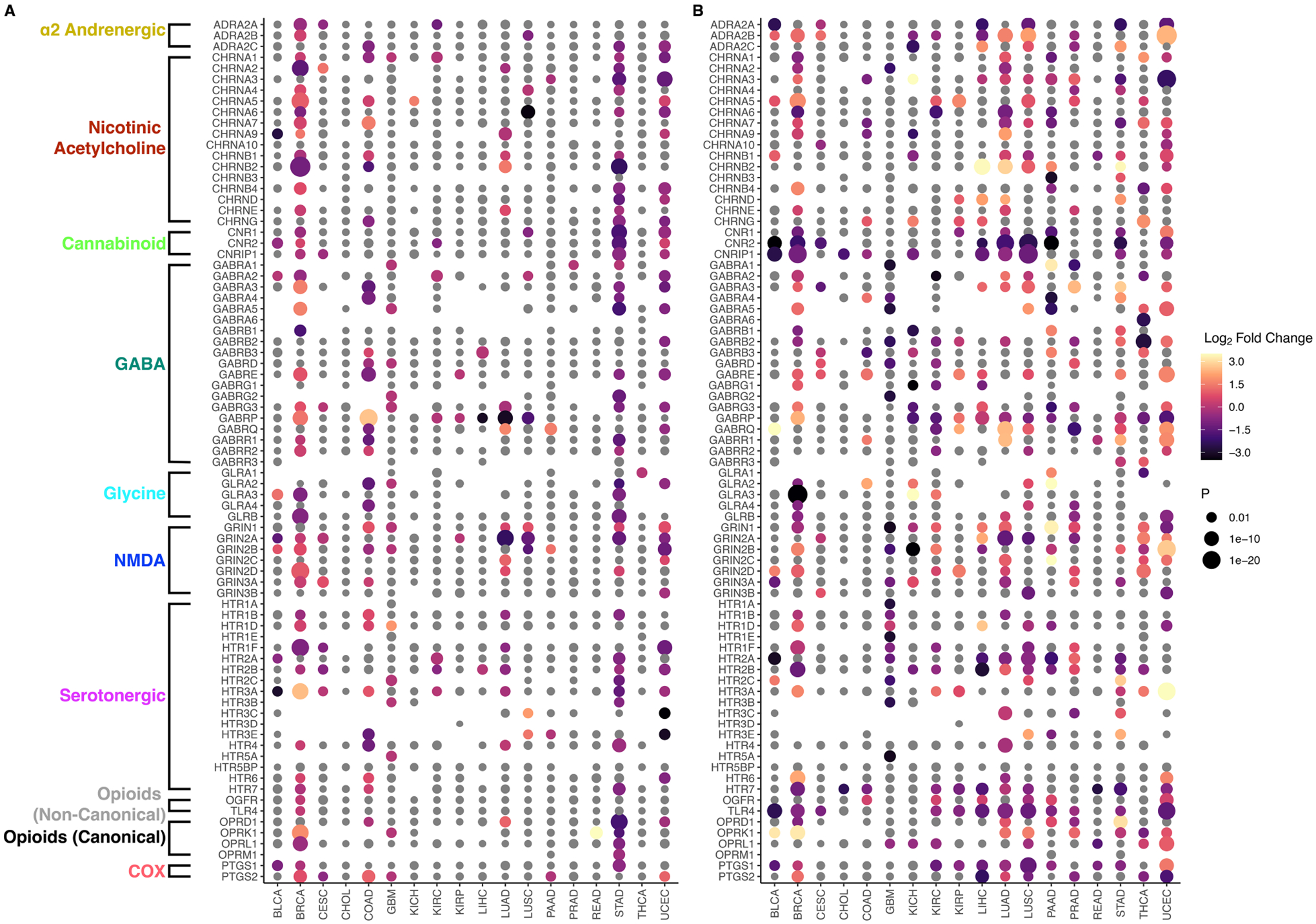
Pan-cancer correlation of TMB and FGA with anesthetic/analgesic target receptor gene expression: Dot plots for differential gene expression of target receptor genes (vertical axis) separated by TCGA cancer type (*x*-axis) for high vs. low TMB **(A)** and FGA **(B)** Greyed out dots represent non-significant differential expressions. For gene-cancer type pairs without a dot, DESeq2 did not produce a valid *p*-value or not enough expression of the gene was found.

Dot plots showing differential target receptor gene expression for all 18 TCGA cancer types for both TMB and FGA are displayed in Figure 4. For TMB (Figure 4A), the genes that are upregulated significantly most frequently are *GRIN1* (5 cancer types), *CHRNA5* (4), *PTGS2, HTR3A, HTR1D, GRIN3A, GRIN2D*, and *GRIN2B* (3). Similarly, the genes that are downregulated significantly the most frequently are *HTR2B, GRIN2A* (7), *HTR3A, GABRP, GABRG3, GABRA2*, and *CHRNA1* (5). With the same determination for FGA (Figure 4B), the upregulated genes are *GABRE* (8), *OPRK1, GRIN2D, GABRA3, CHRNA5* (7), *HTR3A, GRIN1, ADRA2B*, and *GABRQ* (6), and the downregulated genes are *TLR4* (12), *HTR7* (11), *CNRIP1* (10), *PTGS1*, *CNR2* (9), *HT2RB* (8), *HT2RA*, and *GABRP* (7). When comparing pan-cancer if genes were alternatively down- and upregulated by increasing TMB or FGA, 7 TCGA cancer types were found to have at least 5 significant genes that were common between the TMB differential expression experiment and the FGA differential expression experiment. The 7 types were BRCA, COAD, GBM, LUAD, LUSC, STAD, and UCEC. For these types, GBM had 0/10 genes (0%) swap, BRCA had 6/34 (17.6%), LUAD had 3/15 (20%), LUSC had 2/5 (40%), STAD had 12/21 (57.1%), UCEC had 17/21(81.0%), and COAD had 8/8 (100%) common genes swap whether they were up- or downregulated between high/low TMB/FGA. Differential expression results for all cancer types may be found in the Supplementary Data File.

### TME immune composition and target receptor gene expression varies with TMB and FGA

**FIGURE 5:**
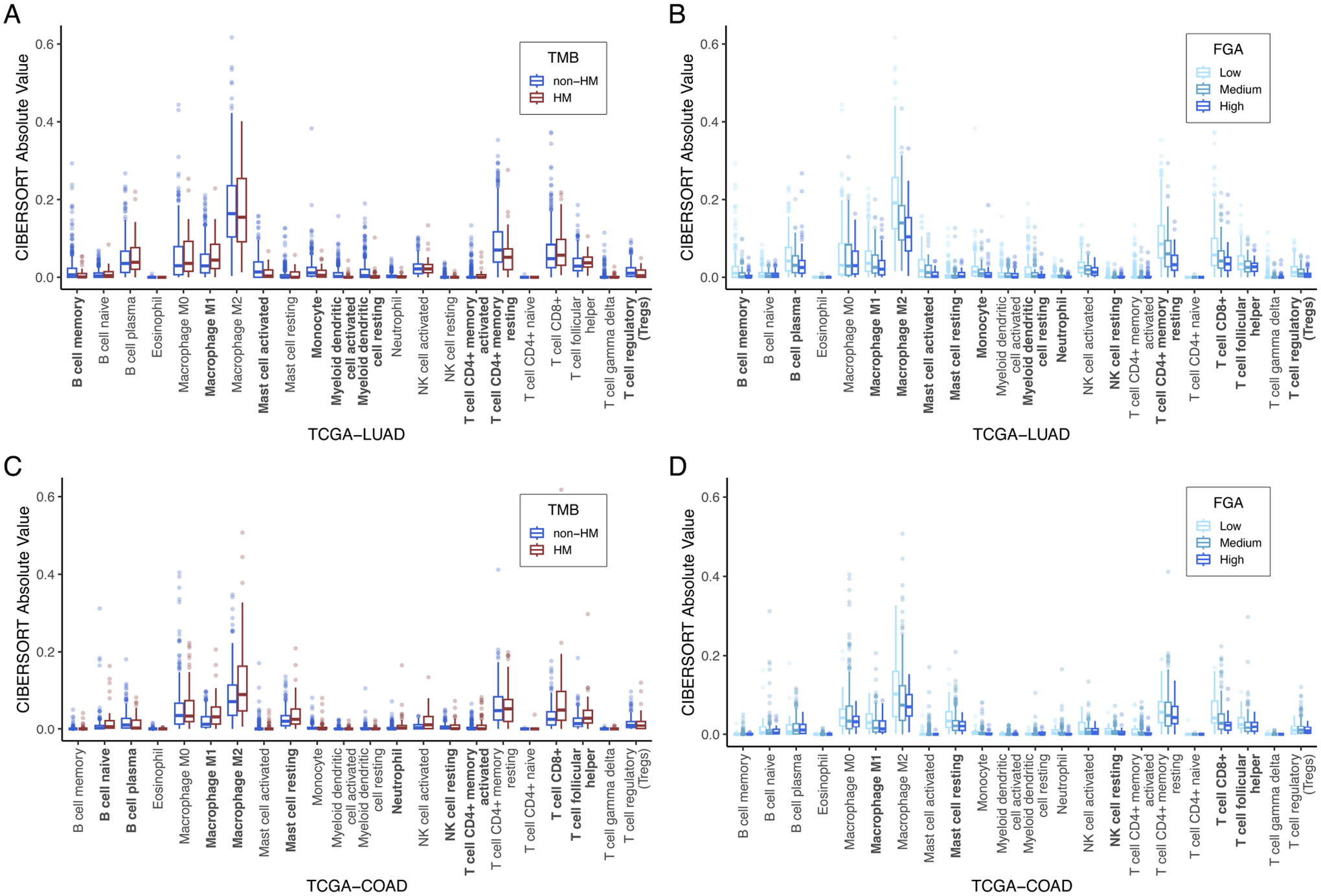
TME immune cell composition correlation with TMB and FGA: Computational estimates for 22 immune cell-type populations (measured by CIBERSORT) are plotted as a function of high vs. low TMB **(A)** and FGA **(B)** for TCGA-LUAD and for TCGA-COAD **(C,D)**. Bolded *x*-axis labels indicate significantly different immune cell-type populations by the Kruskal-Wallis test. CIBERSORT, cell-type identification by estimating relative subsets of ribonucleic acid transcripts; FGA, fraction genome altered; TCGA-COAD, The Cancer Genome Atlas colon adenocarcinoma cohort; TCGA-LUAD, The Cancer Genome Atlas lung adenocarcinoma cohort; TMB, tumor mutational burden.

Consistent with existing literature (45–47), variation in immune composition correlated with TMB and FGA. TCGA-LUAD immune composition is shown in Figures 5A,B for TMB and FGA variation, respectively, while TCGA-COAD immune composition is shown in Figures 5C,D, respectively. For hypermutated tumors (high TMB), most cancer types showed a significant increase in CD8+ T-cells and macrophages, while noting that many subtypes showed exception to this trend. For high FGA, CD8+ T-cells, macrophages (all subtypes but specifically M2 macrophages), and CD4+ memory resting T-cells are significantly decreased. One exception to this rule is TCGA-PRAD (figures and data for all cancer types can be found in Supplementary Data).

**FIGURE 6:**
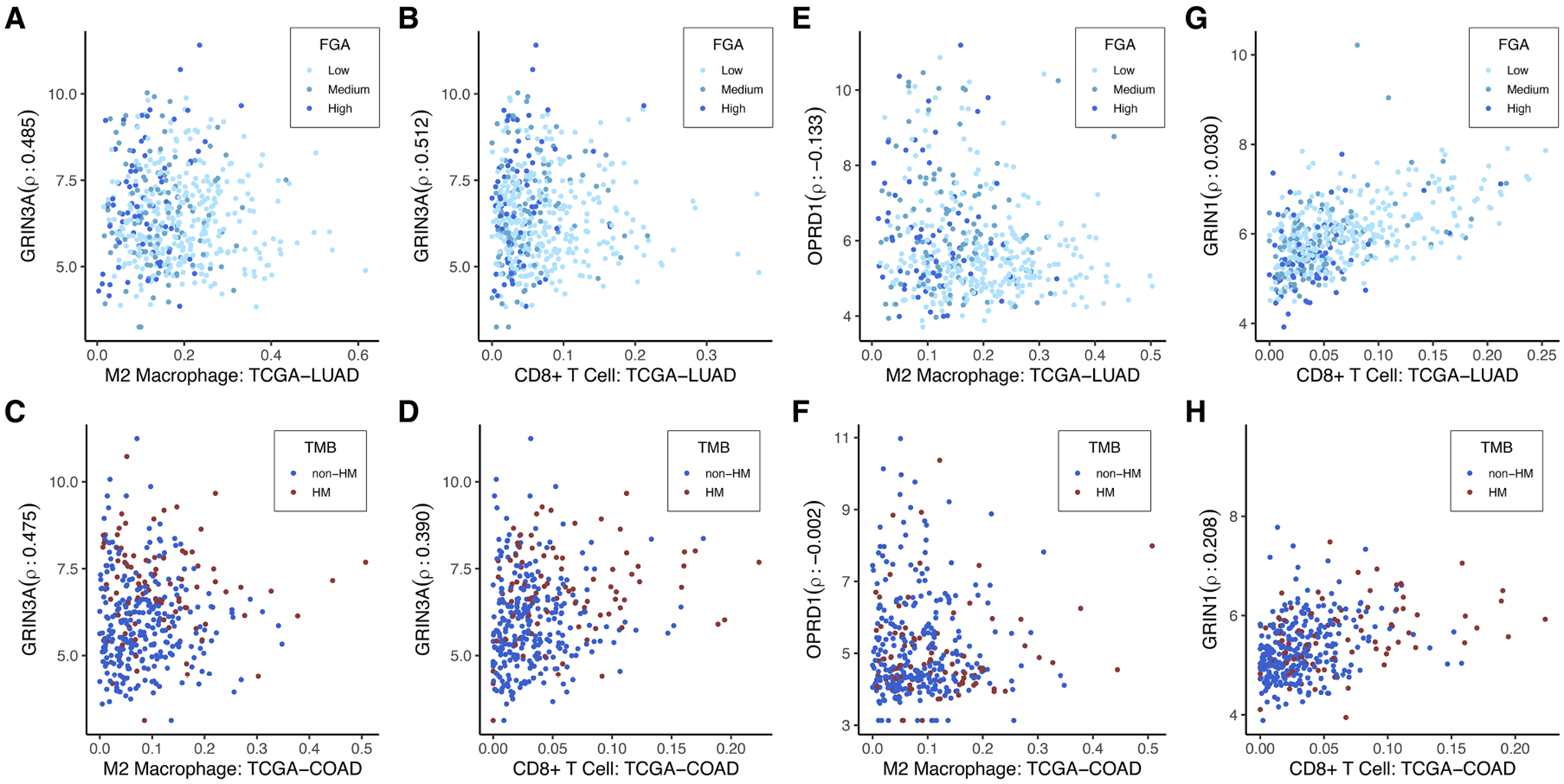
Immune cell-type specificity of anesthetic/analgesic target receptor genes: Correlation of expression of highlighted genes with specific immune cell types for TCGA-LUAD and TCGA-COAD [*GRIN3A* **(A**–**D)**, *OPRD1* **(E**–**F)**, *GRIN1* **(G**–**H)**]. Outliers on the x-axis that were greater than 2 interquartile ranges above the upper quartile were removed to allow for better visualization. These were still included in the correlation calculations. TCGA-LUAD, The Cancer Genome Atlas lung adenocarcinoma cohort; TCGA-COAD, The Cancer Genome Atlas colon adenocarcinoma cohort; FGA, fraction genome altered; HM, hypermutator (high TMB); TMB, tumor mutational burden.

The anesthetic/analgesic target receptor genes found to be significantly correlated with TMB or FGA in TCGA-COAD and TCGA-LUAD were assessed for correlation with the 22 immune cell populations in each cancer type. Correlation was assessed using Spearman’s correlation and *p*-values were computed using the permutation test from the R package *coin* (48). Comparison was done using Spearman’s correlation so as to make no assumption of the linearity when analyzing the correlation of immune cell composition with the receptor genes. Data is provided in Supplementary Data File for the correlation between all significant genes and immune types with resulting *p*-values that were adjusted for multiple hypothesis testing within each immune cell type for each cancer type. Sample correlation plots are displayed in Figure 6. Data is shown for the expression of 3 example genes that were significantly differentially expressed for the TCGA-COAD TMB and TCGA-LUAD FGA categories (*GRIN3A, OPRD1*, and *GRIN1*). There is no clear functional significance at this time for selection of these genes; they are used to illustrate how differential expression may be affecting different immune cells in similar or different ways in various tumor types. These were compared to the presence of M2-type macrophages and CD8+ T-cells, also significantly different when comparing TCGA-COAD TMB and TCGA-LUAD FGA categories. As shown, *GRIN3A* is highly expressed in both TCGA-LUAD and TCGA-COAD across a wide variety of immune cells (Figures 6A–D, *p* < 1e-11 for all 4). However, *OPRD1* shows a significant decreasing expression (*p* = 0.006) in TCGA-LUAD with an increase in M2 macrophages, but no change in expression in TCGA-COAD (*p* = 0.97). In contrast, *GRIN1* shows no significant change in expression based on CD8+ T-cell level in TCGA-LUAD (*p* = 0.58), but an increase in expression is found with an increase in CD8+ T-cells in TCGA-COAD (*p* = 0.0004).

**FIGURE 7:**
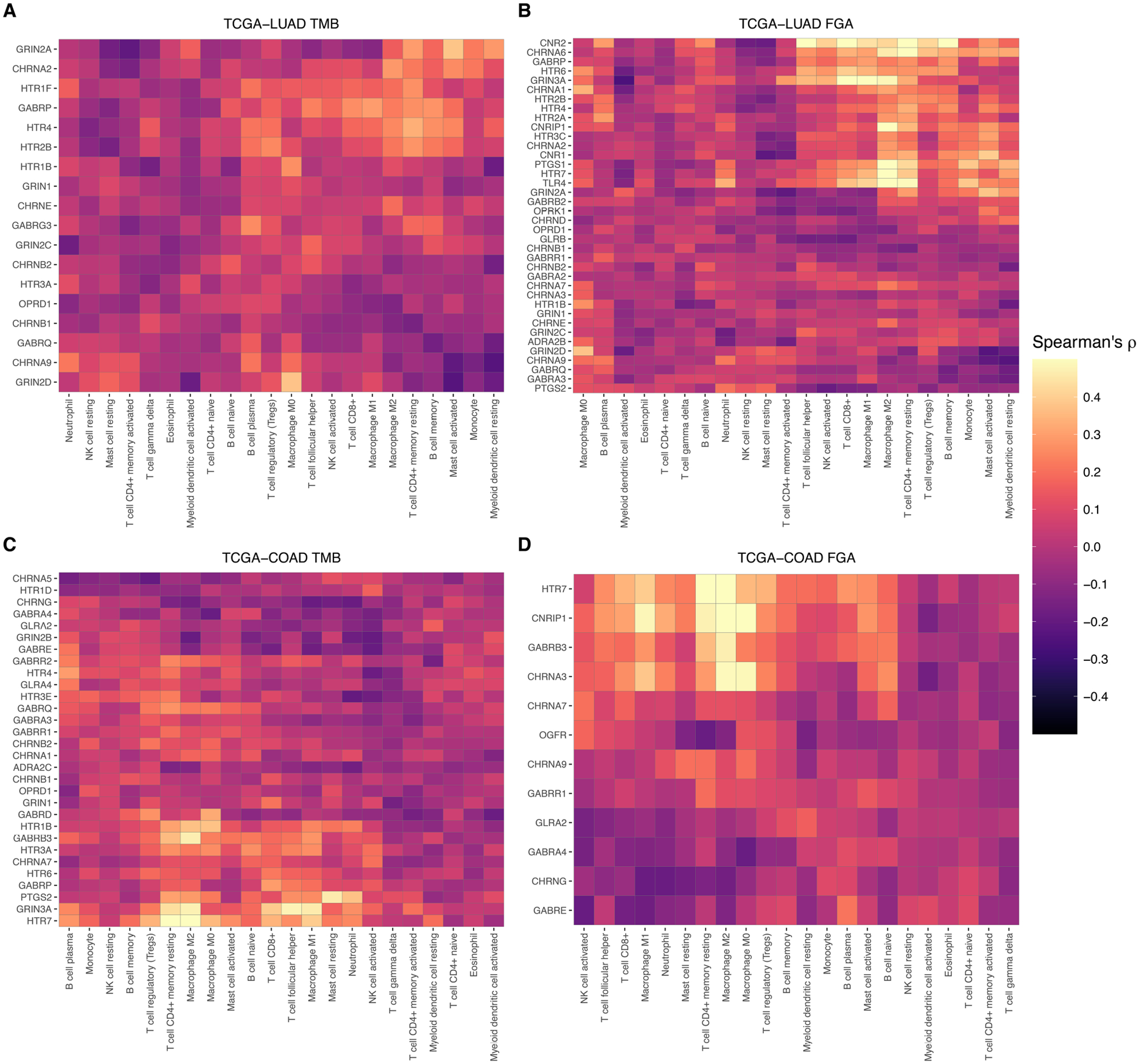
Immune cell-type specificity of anesthetic/analgesic target receptor gene expression: Heatmaps illustrating correlations of target receptor gene expression and immune cell type in TCGA-LUAD for receptors correlated with TMB **(A)** and FGA **(B)** and in TCGA-COAD **(C,D)**. FGA, fraction genome altered; HM, hypermutator (high TMB); TCGA-COAD, The Cancer Genome Atlas colon adenocarcinoma cohort; TCGA-LUAD, The Cancer Genome Atlas lung adenocarcinoma cohort.

Heatmaps displaying Spearman’s rho for TCGA-LUAD and TCGA-COAD are shown for the significant genes for TMB and FGA in Figure 7. These plots are both clustered by cell type and gene expression. As evidenced, specific genes that may be descriptive of a broader cell type such as *GRIN3A* or *HTR7* for macrophages are generally upregulated in samples with estimated higher macrophages, while other genes that may be differentially expressed in a cancer type due to underlying genomic events in the tumor such as hypermutation or genomic alteration can cause significant differences to appear.

## Discussion

The aim of this study is to begin to develop a precision approach to immunomodulation by anesthetic and analgesic drugs to affect outcomes in cancer. At a molecular level, the idea of immunomodulation translates to action on lymphocytes in the TME (i.e., TILs), and so analogy is made to immunotherapy, in particular ICI, which also act on TILs to enhance immune response against tumor cells. We reason that anesthetic/analgesic immunomodulation would be sensitive to the level of TILs in the TME just as ICI are, and would therefore correlate with tumor immunogenicity, captured by the TMB biomarker. Following the analogy made to ICI, we also explored FGA, associated with ICI resistance, even though its relation to TME immune composition is not as defined.

The first major finding of this paper is that there is in fact evidence for TMB and FGA modifying the dose-response curves for analgesics and survival: specifically, TMB and FGA modify the opioid-OS association in LUAD, TMB modifies the ketamine-RSS association in LUAD, and TMB modifies the opioid-recurrence association in COAD. This fundamentally supports the hypothesis that these drugs may have effects on recurrence and survival through immunomodulation. Further, the magnitude of this effect may depend on individual patient tumor immunogenicity and TILs infiltration.

The second major finding is that gene expression of common anesthetic/analgesic drugs correlates with TMB (and FGA) and that the pattern of this relationship (and the immune cell types correlated with receptor expression) depends on specific cancer type. Just as ICI target receptor PD-1 is expressed on TILs (and both target receptors PD-1 and PDL-1 and are themselves biomarkers of response) (49), the expression levels of various anesthetic/analgesic target receptors appear to vary with immunogenicity and may account at least partially for the observed drug interactions with TMB and FGA (Table 1). Regarding opioids, the canonical receptor *OPRD1* is correlated with TMB for both LUAD and COAD, though in opposite directions (upregulated in LUAD and downregulated in COAD) (Figures 2, 3). This is consistent with TMB modification of the opioid survival/recurrence curves for both cancers as well as the difference in directionality of opioid effect (pro-tumor in LUAD, anti-tumor in COAD) (Figures 1E,I). Upregulation of *OPRD1* and *OPRK1* with increasing FGA (Figure 2) is consistent with FGA modification of the opioid-survival curves in LUAD (Figure 1G). Increased TMB and FGA are both correlated with upregulation of OPRD1 in LUAD, consistent with both TMB and FGA magnifying the pro-tumor opioid-survival association in LUAD (Figures 1E,G). Regarding ketamine, TMB modification of the dose-RSS curves in LUAD (Figure 1C) is consistent with its correlation with *GRIN* gene expression (Figure 2). By contrast, ketorolac’s target receptors *PTGS1* and *PTGS2* are not correlated with TMB in LUAD (Figure 4), consistent with TMB not modifying the dose-survival curves (Figure 1D); in this way ketorolac can be seen as a negative control compared with the other drugs.

The results in Figure 4 are consistent with data in the literature as well. Variation of target receptor expression with cancer type is consistent with previous work looking more generally at opioid receptor genes and tumor gene expression (not specifically immune gene expression) (50). It is notable that serotonergic receptor differential expression correlates with TMB and FGA in a variety of cancers (Figure 4), considering recent evidence that these drugs may have oncological effects (51) and representation in TCGA-LIHC specifically (Figure 4) is consistent with recent clinical evidence in hepatocellular carcinoma (52).

Interestingly, high TMB and FGA modify the opioid dose to survival response in the same direction in LUAD, whereas high TMB and FGA predict opposite responses for ICI. This does not contradict the underlying hypothesis that opioid immunomodulation acts on the same TILs substrate as immunotherapy but rather highlights the possibility that the specific pathways regulated may be different for different drugs, which may be affected differently with increase in copy number alteration. Similarly, increased TMB magnifies the pro-tumor opioid-OS association in TCGA-LUAD while reducing the anti-tumor ketamine-RSS association. This likely reflects different pathways of interaction between these specific drugs and TILs, not to mention differences in direct on-tumor effects of drugs (vs. drug regulation of TILs action to affect tumor). These findings underscore that immunomodulation is nuanced, a finding consistent with recent epidemiological evidence that opioids may decrease ICI efficacy (53–56) while evincing [in the case of COAD or triple-negative breast cancer (TNBC)] anti-tumor effect.

We recently proposed a mechanism for such opioid-ICI interaction, based on analysis of gene expression and drug-induced transcriptomic signatures, suggesting that opioids and ICI regulate a common gene network in CD8+ T-cells in the TME, but in opposite directions (57). Drug interaction with ICI may depend on pathways separable from drug immunomodulation more generally with possibly opposing effects on survival. This may partially explain our findings that ketamine can be simultaneously anti-tumor (i.e., lower recurrence risk) due to direct on-tumor effects, that this may be mitigated through ketamine interaction with TILs (i.e., reducing the RSS improvement with increasing TMB), and also be neutral with respect to ICI efficacy (predicted in the mechanistic modeling of drug-ICI interaction).

Limitations of this study include the following. First, the clinical data related to opioids and ketamine derives from intraoperative dosing. While it is reasonable that these effects extend (and are perhaps even more pronounced) in chronic dosing for cancer pain, this remains speculative. Second, at present we have limited data relating drugs, outcomes, and TMB and FGA. Consequently, we have focused on opioids, ketamine, and ketorolac in analysis of the clinical data supporting TMB and FGA modification of drug-survival dose-response, while the target receptor exploration is broader. Ideally, we would have the requisite data to similarly analyze other anesthetic/analgesic agents for dose-response and TMB/FGA modification. Similarly, the clinical data is limited to two cancer types (LUAD and COAD). A third limitation is that we have employed bulk gene expression data to make inferences about immune cells specifically. Ideally, we would demonstrate these relationships in single-cell data derived from specific immune cell populations in the TME (in combination with the bulk data, which is needed for calculation of TMB and FGA). This data, however, is limited and not sufficient for pan-cancer analysis. Furthermore, it is often derived from only a few patients, whereas elucidating correlation with TMB and FGA requires larger patient samples. Future research could employ single-cell immune cell data to corroborate expression of target receptors on TILs, as we have already done for TNBC (9). Finally, while the exploratory analysis of target receptor gene expression (and correlation with TMB/FGA) suggests an underlying mechanism to explain individual patient variation in immunomodulation by anesthetic/analgesic drugs, this is beyond the scope of the present work. The possible regulation by these receptors of specific downstream oncogenic pathways is not elucidated here, nor is the relation between expression level and degree of regulation, both of which are necessary to formulate a mechanism for variation of immunomodulation by the drugs to affect outcomes. Additionally, the current exploration focuses specifically on immunomodulation, while noting that these drugs may have direct on-tumor effects as well.

Future research should aim to understand whether TMB and other markers related to immunogenicity and TILs infiltration can modify relationships between other anesthetic/analgesic drugs and outcomes and in other cancer types, whether those drugs are dosed in the intraoperative or chronic settings. Models for studying immunotherapy are well described and may be applicable here; these include syngeneic mouse tumor models, genetically engineered mouse models (GEMMs), cell line-derived xenografts (CDX), patient-derived xenografts (PDX), humanized mouse models, and tumor organoids/spheroids (58). Ultimately, prospective validation is important, which could definitively establish these markers as predictive. As the cost of acquiring omics data continues to decrease (59), its incorporation in prospective studies will become more feasible. Throughout, it is imperative to look at specific cancer types and subtypes individually, as immunogenicity fundamentally varies between cancer types (60). Our results argue strongly for a precision approach to understanding immunomodulation by anesthetic/analgesic drugs in cancer to inform future research and ultimately for application at the clinical level.

## Supplementary Material

Supplementary Material

Supplementary Data File 1

Supplementary Data File 2

Supplementary Data File 3

The Supplementary Material for this article can be found online at: https://www.frontiersin.org/articles/10.3389/fanes.2024.1464004/full#supplementary-material

## Figures and Tables

**TABLE 1:** Comparison of clinical (drug) and transcriptomic (receptor gene) interactions with TMB and FGA.

Drug	Cancer type	Outcome	Dose-response	TMB/FGA interaction	Modification of dose-response	Target receptor gene	Correlation with TMB/FGA
Opioids	LUAD	OS	Pro-tumor	TMB	Increased pro-tumor	*OPRD1*	Upregulated
				FGA	Increased pro-tumor	*OPRD1*, *OPRK1*	Upregulated
	COAD	Recurrence risk	Anti-tumor	TMB (dMMR)	Increased anti-tumor	*OPRD1*	Downregulated
Ketamine	LUAD	RSS	Anti-tumor	TMB	Reduced anti-tumor	*GRIN1*	Upregulated
						*GRIN2A*	Downregulated
						*GRIN2C*	Upregulated
						*GRIN2D*	Upregulated
Ketorolac	LUAD	RSS	Anti-tumor	None	None	*PTGS1*	Unchanged
						*PTGS2*	Unchanged

COAD, colon adenocarcinoma; FGA, fraction genome altered; LUAD, lung adenocarcinoma; MMR, DNA mismatch repair (dMMR, MMR-deficient); OS, overall survival; RSS, recurrence-specific survival; TMB, tumor mutational burden.

## Data Availability

The data analyzed in this study is subject to the following licenses/restrictions: TCGA data is publicly available. Data in the MSK patient cohorts is not publicly available. Requests to access these datasets should be directed to mincerj@mskcc.org.
