# Supplementary material for: Towards a precision approach to anesthetic/analgesic immunomodulation in cancer
Source: Front Anesthesiol. Author manuscript; Available in PMC 2025 Sep 4. (PMC12407147; doi:10.3389/fanes.2024.1464004)
Supplement: Supplementary Material [file NIHMS2100776-supplement-Supplementary_Material.docx]

SUPPLEMENTARY MATERIALS

SUPPLEMENTARY FIGURES

Supplementary Fig. 1. Predicted 5-year overall survival estimates across a range of opioid doses for model patients with derived FGA values (Fig. 1G with 95% confidence bands added).

Supplementary Fig. 2. TMB distributions for the 18 TCGA cancer types.

Supplementary Fig. 3. FGA distributions for the 18 TCGA cancer types.

Supplementary Fig. 4. Volcano plots for high vs medium FGA for TCGA-COAD and TCGA-LUAD.

SUPPLEMENTARY TABLES

Supplementary Table 1. TCGA sample counts by cancer type.

Supplementary Table 2. Anesthetic/analgesic target receptor genes.

Supplementary Table 3. TMB breakpoints for all TCGA types.

Supplementary Table 4. FGA breakpoints for all TCGA types.

**Supplementary Figure 1**

**Supplementary Figure 2**

**Supplementary Figure 3**

**Supplementary Figure 4**

**Supplementary Table 1**

| TCGA Cancer Type | Count |
| --- | --- |
| BLCA | 400 |
| BRCA | 1039 |
| CESC | 284 |
| CHOL | 36 |
| COAD | 372 |
| GBM | 150 |
| KICH | 64 |
| KIRC | 392 |
| KIRP | 274 |
| LIHC | 356 |
| LUAD | 494 |
| LUSC | 480 |
| PAAD | 170 |
| PRAD | 483 |
| READ | 139 |
| STAD | 369 |
| THCA | 483 |
| UCEC | 503 |
| Total | 6488 |

**Supplementary Table 2**

| Gene | Class | Gene | Class | Gene | Class |
| --- | --- | --- | --- | --- | --- |
| ADRA2A | alpha-2 adrenergic | GABRA6 | GABAAR | HTR1A | 5-HT (GPCR) |
| ADRA2B | alpha-2 adrenergic | GABRB1 | GABAAR | HTR1B | 5-HT (GPCR) |
| ADRA2C | alpha-2 adrenergic | GABRB2 | GABAAR | HTR1D | 5-HT (GPCR) |
| CHRNA1 | nAChR | GABRB3 | GABAAR | HTR1E | 5-HT (GPCR) |
| CHRNA10 | nAChR | GABRD | GABAAR | HTR1F | 5-HT (GPCR) |
| CHRNA2 | nAChR | GABRE | GABAAR | HTR2A | 5-HT (GPCR) |
| CHRNA3 | nAChR | GABRG1 | GABAAR | HTR2B | 5-HT (GPCR) |
| CHRNA4 | nAChR | GABRG2 | GABAAR | HTR2C | 5-HT (GPCR) |
| CHRNA5 | nAChR | GABRG3 | GABAAR | HTR3A | 5-HT (ionotropic) |
| CHRNA6 | nAChR | GABRP | GABAAR | HTR3B | 5-HT (ionotropic) |
| CHRNA7 | nAChR | GABRQ | GABAAR | HTR3C | 5-HT (ionotropic) |
| CHRNA9 | nAChR | GABRR1 | GABAAR | HTR3D | 5-HT (ionotropic) |
| CHRNB1 | nAChR | GABRR2 | GABAAR | HTR3E | 5-HT (ionotropic) |
| CHRNB2 | nAChR | GABRR3 | GABAAR | HTR4 | 5-HT (GPCR) |
| CHRNB3 | nAChR | GLRA1 | GlyR | HTR5A | 5-HT (GPCR) |
| CHRNB4 | nAChR | GLRA2 | GlyR | HTR5BP | 5-HT (GPCR) |
| CHRND | nAChR | GLRA3 | GlyR | HTR6 | 5-HT (GPCR) |
| CHRNE | nAChR | GLRA4 | GlyR | HTR7 | 5-HT (GPCR) |
| CHRNG | nAChR | GLRB | GlyR | OGFR | opioid - noncanonical |
| CNR1 | cannabinoid | GRIN1 | NMDA | OPRD1 | opioid - canonical |
| CNR2 | cannabinoid | GRIN2A | NMDA | OPRK1 | opioid - canonical |
| CNRIP1 | cannabinoid | GRIN2B | NMDA | OPRL1 | opioid - canonical |
| GABRA1 | GABAAR | GRIN2C | NMDA | OPRM1 | opioid - canonical |
| GABRA2 | GABAAR | GRIN2D | NMDA | PTGS1 | COX |
| GABRA3 | GABAAR | GRIN3A | NMDA | PTGS2 | COX |
| GABRA4 | GABAAR | GRIN3B | NMDA | TLR4 | opioid - noncanonical |
| GABRA5 | GABAAR |  |  |  |  |

**Supplementary Table 3**

| Cancer Type | Breakpoint | TMB | Chosen Break |
| --- | --- | --- | --- |
| BLCA | 349 | 13.9666667 | Yes |
| BLCA | 390 | 22.7666667 | No |
| BRCA | 765 | 2.13333333 | Yes |
| BRCA | 1013 | 6.13333333 | No |
| CESC | 236 | 5.7 | Yes |
| CESC | 276 | 17.8666667 | No |
| CHOL | 22 | 1.63333333 | No |
| CHOL | 35 | 4.7 | Yes |
| COAD | 266 | 4.93333333 | No |
| COAD | 299 | 5.93333333 | Yes |
| GBM | 332 | 2.63333333 | Yes |
| GBM | 378 | 6.33333333 | Yes |
| KICH | 45 | 0.76666667 | No |
| KICH | 62 | 1.56666667 | Yes |
| KIRC | 68 | 0.76666667 | No |
| KIRC | 382 | 3.26666667 | Yes |
| KIRP | 236 | 3.66666667 | Yes |
| KIRP | 263 | 5.96666667 | No |
| LIHC | 313 | 4.66666667 | Yes |
| LIHC | 348 | 7.6 | No |
| LUAD | 121 | 2.33333333 | No |
| LUAD | 515 | 23.5666667 | Yes |
| LUSC | 90 | 4.8 | No |
| LUSC | 425 | 15.1333333 | Yes |
| PAAD | 146 | 1.76666667 | No |
| PAAD | 171 | 2.66666667 | Yes |
| PRAD | 385 | 1.26666667 | No |
| PRAD | 470 | 2.26666667 | Yes |
| READ | 126 | 4.76666667 | Yes |
| READ | 133 | 10.9666667 | Yes |
| STAD | 282 | 5.36666667 | No |
| STAD | 325 | 8.26666667 | Yes |
| THCA | 389 | 0.56666667 | No |
| THCA | 466 | 1.16666667 | Yes |
| UCEC | 331 | 9.93333333 | Yes |
| UCEC | 445 | 36.1 | Yes |

**Supplementary Table 4**

| Histology | FGA Break | FGA |
| --- | --- | --- |
| BLCA | 22 | 0.0047 |
| BLCA | 369 | 0.5727 |
| BRCA | 696 | 0.3607 |
| BRCA | 1015 | 0.6698 |
| CESC | 23 | 0.078 |
| CESC | 244 | 0.4112 |
| CHOL | 7 | 0.1498 |
| CHOL | 28 | 0.4619 |
| COAD | 40 | 0.0042 |
| COAD | 393 | 0.465 |
| GBM | 44 | 0.0911 |
| GBM | 511 | 0.3176 |
| KICH | 17 | 0.7694 |
| KICH | 27 | 0.9158 |
| KIRC | 408 | 0.2422 |
| KIRC | 481 | 0.518 |
| KIRP | 219 | 0.2451 |
| KIRP | 254 | 0.4023 |
| LIHC | 40 | 0.088 |
| LIHC | 329 | 0.5099 |
| LUAD | 319 | 0.3168 |
| LUAD | 430 | 0.4939 |
| LUSC | 130 | 0.2713 |
| LUSC | 416 | 0.6161 |
| PAAD | 64 | 0.012 |
| PAAD | 171 | 0.4243 |
| PRAD | 374 | 0.1252 |
| PRAD | 465 | 0.3105 |
| READ | 29 | 0.1588 |
| READ | 147 | 0.5784 |
| STAD | 199 | 0.1513 |
| STAD | 417 | 0.5775 |
| THCA | 365 | 0.0094 |
| THCA | 474 | 0.0835 |
| UCEC | 278 | 0.0888 |
| UCEC | 431 | 0.4314 |
