# Supplementary material for: Towards a precision approach to anesthetic/analgesic immunomodulation in cancer
Source: Front Anesthesiol. Author manuscript; Available in PMC 2025 Sep 4. (PMC12407147; doi:10.3389/fanes.2024.1464004)
Supplement: Supplementary Data File 3 [file NIHMS2100776-supplement-Supplementary_Data_File_3.zip › SupplementaryData_3/immune_comparisons.tar.xz!/immune_comparisons/figures/fga_cibersort_abs_STAD.pdf]

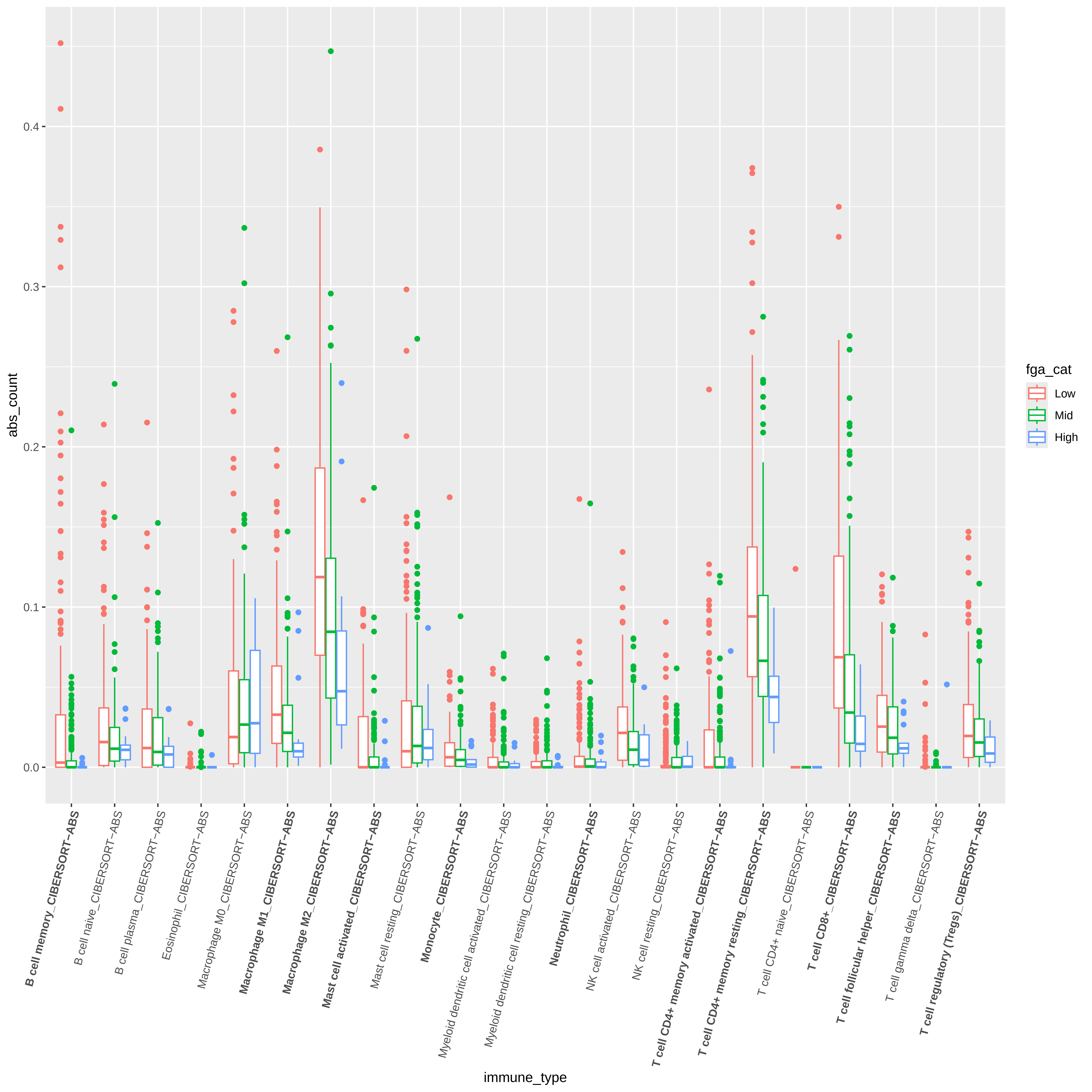

abs_count
0.0
0.1
0.2
0.3
0.4
immune_type
fga_cat
Low
Mid
High
B cell memory_CIBERSORT-ABS
B cell naive_CIBERSORT-ABS
B cell plasma_CIBERSORT-ABS
Eosinophil_CIBERSORT-ABS
Macrophage M0_CIBERSORT-ABS
Macrophage M1_CIBERSORT-ABS
Macrophage M2_CIBERSORT-ABS
Mast cell activated_CIBERSORT-ABS
Mast cell resting_CIBERSORT-ABS
Monocyte_CIBERSORT-ABS
Myeloid dendritic cell activated_CIBERSORT-ABS
Myeloid dendritic cell resting_CIBERSORT-ABS
Neutrophil_CIBERSORT-ABS
NK cell activated_CIBERSORT-ABS
NK cell resting_CIBERSORT-ABS
T cell CD4+ memory activated_CIBERSORT-ABS
T cell CD4+ memory resting_CIBERSORT-ABS
T cell CD4+ naive_CIBERSORT-ABS
T cell CD8+_CIBERSORT-ABS
T cell follicular helper_CIBERSORT-ABS
T cell gamma delta_CIBERSORT-ABS
T cell regulatory (Tregs)_CIBERSORT-ABS
