# Supplementary material for: Towards a precision approach to anesthetic/analgesic immunomodulation in cancer
Source: Front Anesthesiol. Author manuscript; Available in PMC 2025 Sep 4. (PMC12407147; doi:10.3389/fanes.2024.1464004)
Supplement: Supplementary Data File 3 [file NIHMS2100776-supplement-Supplementary_Data_File_3.zip › SupplementaryData_3/immune_comparisons.tar.xz!/immune_comparisons/figures/hm_cibersort_abs_coad.pdf]

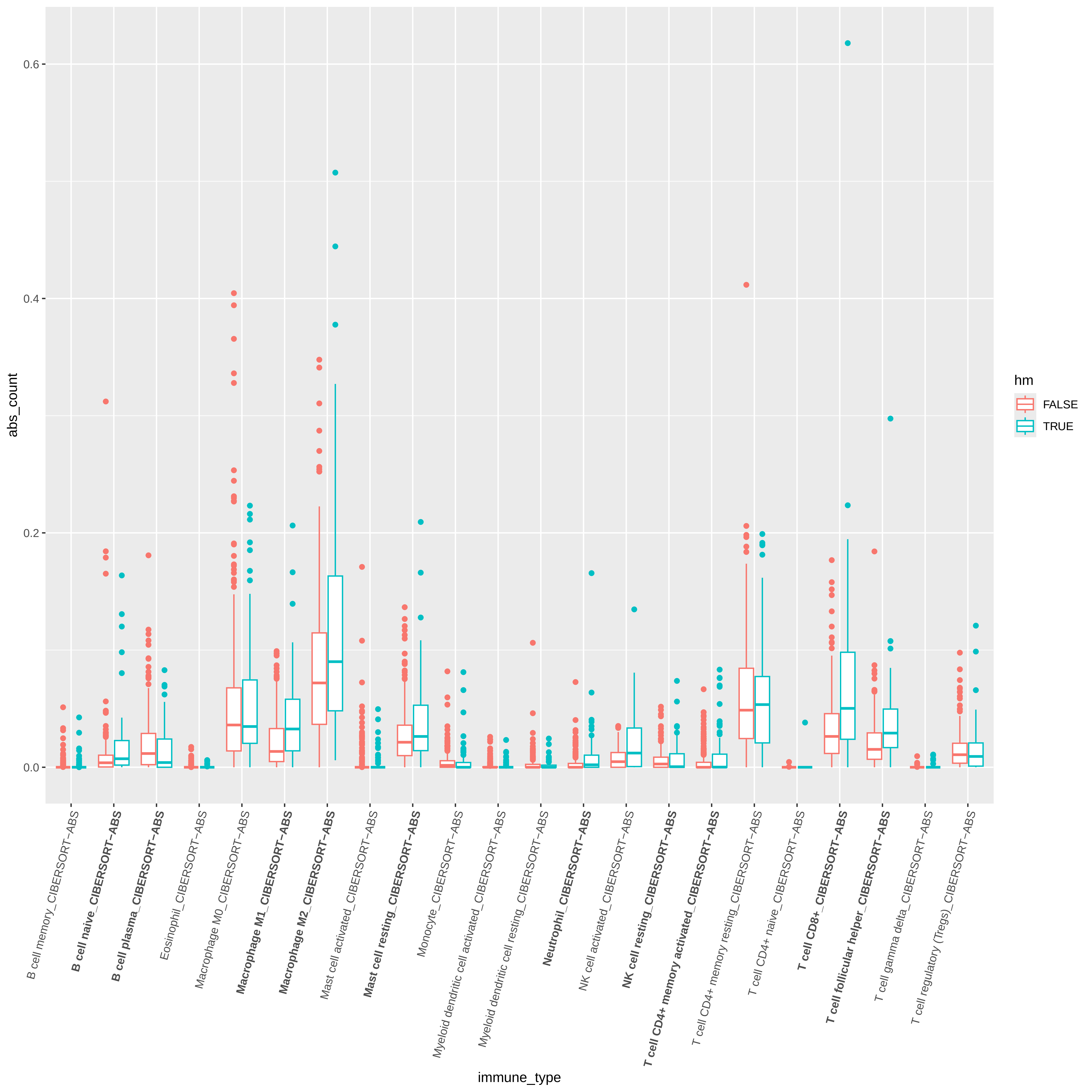

abs_count
0.0
0.2
0.4
0.6
immune_type
hm
FALSE
TRUE
B cell memory_CIBERSORT−ABS
B cell naive_CIBERSORT−ABS
B cell plasma_CIBERSORT−ABS
Eosinophil_CIBERSORT−ABS
Macrophage M0_CIBERSORT−ABS
Macrophage M1_CIBERSORT−ABS
Macrophage M2_CIBERSORT−ABS
Mast cell activated_CIBERSORT−ABS
Mast cell resting_CIBERSORT−ABS
Monocyte_CIBERSORT−ABS
Myeloid dendritic cell activated_CIBERSORT−ABS
Myeloid dendritic cell resting_CIBERSORT−ABS
Neutrophil_CIBERSORT−ABS
NK cell activated_CIBERSORT−ABS
NK cell resting_CIBERSORT−ABS
T cell CD4+ memory activated_CIBERSORT−ABS
T cell CD4+ memory resting_CIBERSORT−ABS
T cell CD4+ naive_CIBERSORT−ABS
T cell CD8+_CIBERSORT−ABS
T cell follicular helper_CIBERSORT−ABS
T cell gamma delta_CIBERSORT−ABS
T cell regulatory (Tregs)_CIBERSORT−ABS
